# Annual acknowledgement of manuscript reviewers

**DOI:** 10.1186/1472-6815-14-1

**Published:** 2014-01-30

**Authors:** Magdalena Morawska

**Affiliations:** 1 BioMed Central, Floor 6, 236 Gray's Inn Road, London WC1X 8HB, UK

## Abstract

**Contributing reviewers:**

The editors of *BMC Ear, Nose and Throat Disorders* would like to thank all of our reviewers who have contributed to the journal in Volume 13 (2013).

## 

Alice Holmes

USA

Andrea Diociaiuti

Italy

Basile Landis

Switzerland

Bayeh Abera

Ethiopia

Bo Engdahl

Norway

Bolajoko O Olusanya

Nigeria

Daisuke Mori

Japan

David Hurst

USA

David Kaylie

USA

Dimitri Renard

France

Ellen Mandel

USA

Francesco Martines

Italy

Franco Ionna

Italy

Hasantha Gunasekera

Australia

Heidi Smith-Vaughan

Australia

Hidenobu Matsuzaki

Japan

Isaac Van Der Waal

Netherlands

Janet Gilsdorf

USA

Jeroen Langereis

Netherlands

Kim Kiely

Australia

Lena Setchanova

Bulgaria

Mahmood Bhutta

UK

Markus Moessner

Germany

Martin Chovanec

Czech Republic

Moncef Berhouma

Tunisia

Monique Smeets

Netherlands

Moses Galukande

Uganda

Nikolaos Nikitakis

Greece

Petros Vlastarakos

UK

Pietro Leocata

Italy

Rafael Llinas

USA

Ravinder Kaur

USA

Stavros Hatzopoulos

Italy

Stefan Hegemann

Switzerland

Steven Nordin

Sweden

Sulyman Alabi

Nigeria

Thomas Beech

UK

## Pre-publication history

The pre-publication history for this paper can be accessed here:

http://www.biomedcentral.com/1472-6815/14/1/prepub

